# Cough and dyspnea during bronchoconstriction: comparison of different stimuli

**DOI:** 10.1186/1745-9974-5-6

**Published:** 2009-06-25

**Authors:** Thais R Suguikawa, Clecia A Garcia, Edson Z Martinez, Elcio O Vianna

**Affiliations:** 1 Department of Medicine, Medical School of Ribeirão Preto, University of S. Paulo at Ribeirão Preto, Brazil; 2 Department of Social Medicine, Medical School of Ribeirão Preto, University of S. Paulo at Ribeirão Preto, Brazil

## Abstract

**Background:**

Bronchial challenge tests are used to evaluate bronchial responsiveness in diagnosis and follow-up of asthmatic patients. Challenge induced cough has increasingly been recognized as a valuable diagnostic tool. Various stimuli and protocols have been employed. The aim of this study was to compare cough and dyspnea intensity induced by different stimuli.

**Methods:**

Twenty asthmatic patients underwent challenge tests with methacholine, bradykinin and exercise. Cough was counted during challenge tests. Dyspnea was assessed by modified Borg scale and visual analogue scale. Statistical comparisons were performed by linear mixed-effects model.

**Results:**

For cough evaluation, bradykinin was the most potent trigger (p < 0.01). In terms of dyspnea measured by Borg scale, there were no differences among stimuli (p > 0.05). By visual analogue scale, bradykinin induced more dyspnea than other stimuli (p ≤ 0.04).

**Conclusion:**

Bradykinin seems to be the most suitable stimulus for bronchial challenge tests intended for measuring cough in association with bronchoconstriction.

## Background

Cough is one of the most common symptoms in asthma patients, although little attention has been paid to its role in asthma diagnosis and follow-up. Some recent studies from Europe have suggested that cough provoked by inhalation challenges may be useful in diagnosing asthma [1,2], and also in evaluating the response to asthma treatment [3]. These studies support the concept that cough could be utilized as a surrogate for bronchoconstriction when studying patients likely to be unable to perform spirometry. However, the relationship between intensity of coughing and level of bronchoconstriction is still a matter of debate.

Sheppard et al studied the relationship between cough and bronchoconstriction caused by inhaled distilled water aerosol in subjects with asthma. Atropine caused inhibition of the water-induced bronchoconstriction, but did not inhibit cough. Their data suggest that water-induced bronchoconstriction involves cholinergic nerves and that water-induced cough is not dependent on bronchoconstriction[4]. On the other hand, Koskela et al showed that direct, indirect, and combined airway challenges are able to provoke cough, but the significance of the cough response differs considerably among the challenge stimuli[2].

Therefore, the utility of cough during bronchial challenges in diagnosing asthma may depend on the stimulus. In the present study, we hypothesized that bradykinin causes more cough and dyspnea. Bradykinin is thought to be an indirect stimulus, i.e., causes airflow limitation by an action on cells other than the effector cells (smooth muscle). Possible mechanisms by which bradykinin may cause bronchoconstriction involve the stimulation of sensory nerves to induce smooth muscle contraction via neural reflex pathways and this may contribute to cough stimulus in asthma [5]. The aim of this study was to compare cough and dyspnea intensity induced by bradykinin, methacholine, and exercise challenge.

## Methods

### Subjects

Asthma was defined by clinician diagnosis [6]. We recruited asthmatics with history of symptoms induced by exercise that were studied during a clinically stable period, without symptoms of upper respiratory tract infection for at least six weeks prior to the study. Exclusion criteria were: smoking, other pulmonary disease, pregnancy, use of medication other than bronchodilator or inhaled steroids, inability to perform the exercise challenge, incapacity to understand the protocol, including illiteracy. Long-acting β_2_-agonist was withheld for at least 24 hours, and short-acting β_2_-agonist was withheld for 12 hours before evaluations. Moreover, the time interval between the last dose of bronchodilator and the challenge test was established to be the same before all tests. All subjects gave informed consent to this Institutional Review Board approved protocol.

### Study design

Three challenge tests were performed on three different days, at the same time of day, at least 48 hours apart. Tests sequence was randomly determined. Subjects had their coughs counted during every challenge test and were requested to rate discomfort associated with the act of breathing one minute before every FEV_1 _maneuver during all challenge tests. Patients registered dyspnea intensity in a VAS and answered, according to modified Borg scale.

### Assessment of cough

A cough is a reflex act with an explosive expiration. The three phases of a cough are: 1) a deep inspiration; 2) compression of air in the lungs and airways by forceful concentration of the expiratory muscles coupled with closure of the glottis and opening of the larynx; 3) sudden explosive expiration followed by narrowing of the glottis and return of the larynx to its normal inspiratory position [7]. We counted every phase 3 as one cough. This counting was performed during two minutes before spirometry (baseline evaluation) and during two minutes before every FEV_1 _maneuver during inhalation challenge tests. During exercise challenge test, cough was counted before and after exercise (during all recovering time). The act of clearing the throat was not considered as a cough. The same technician counted cough all over the study, in a quiet and calm environment, without performing other tasks.

### Assessment of perception of dyspnea

Subject was free to interpret respiratory discomfort in any way he or she felt appropriate, and no further instructions were given. The subject rated the intensity of symptom on the modified Borg scale, a scale numbered 0 to 10. These are tagged to descriptive phrases, describing increasing intensities of asthma sensations, and subjects were not restricted to whole numbers[8]. Visual analogue scale was an horizontal straight line (10 cm) labeled "no breathlessness at all" (0 cm) at one end and "the most extreme breathlessness ever experienced" (10 cm) at the other, whereby equal distances are meant to represent equal severities of breathlessness [9,10]. During tests, subjects were blinded to their lung function response and to previous dyspnea scores.

### Inhalation challenge tests

Methacholine and bradykinin challenge tests were performed following the same protocol, according to a standardized tidal breathing method. For safety reasons, baseline FEV_1 _≥ 50% of predicted value was requisite to start challenge tests. Acetyl-β-methylcholine chloride (Sigma-Aldrich, Saint Louis, MO, USA) and tri-acetate of bradykinin in normal phosphate-buffered saline solution were aerosolized by a DeVilbiss 646 nebulizer (Sunrise Medical HHG Inc, Somerset, PA, USA) during tidal breathing for two minutes, driven by a computer-activated dosimeter (Koko Digidoser System, PDS Instrumentation, Inc., Louisville, CO, USA). Phosphate-buffered saline solution was inhaled first, followed by test solution in two-fold increasing concentrations (0.06 to 16 mg/ml). Measurements of FEV_1 _were made using the Koko Spirometer before test and two minutes after every inhalation. Cough was counted during these two-minute intervals. The challenge test was discontinued if FEV_1 _dropped 20% or more from baseline. The provocative concentration of methacholine or bradykinin resulting in a 20% fall in FEV_1 _(PC_20 _MCh or PC_20 _BK, respectively) was calculated by linear interpolation of dose-response curves [11].

### Exercise challenge test

After one minute of light exercise on the inclined (10°) treadmill, the speed was quickly increased to achieve 80% of the maximum predicted heart rate; this speed was then maintained for six minutes. Standard measurements of spirometry were obtained before, immediately after and 5, 10, 15, 20, 30 and 45 minutes after exercise. Cough was counted before and after exercise (during all the recovering time). The maximum percentage of change from baseline was calculated as 100 × the decrease in FEV_1_/baseline FEV_1 _[12]. With the aid of air conditioning apparatus, the exercise laboratory temperature and relative humidity were kept at 20° to 22°C and 50% to 55%, respectively.

### Statistical analysis

Since the response variables are assumed to be continuous, linear mixed-effects models were used to allow for dependencies between measurements on the same patient. These models were used to verify the effect of challenge tests on dyspnea (Borg and VAS) and on cough [13], considering that each patient underwent all the three different tests. We assume that these models have normally distributed residual with mean zero and constant variance. Like this, the distribution of residuals was graphically verified and when it was not compatible with this presupposition, new models were adjusted with logarithms transformation. Statistical interaction was examined by including the independent variables and their cross-product term in the model. Age, gender, atopy status, body mass index, FEV_1_, baseline FEV_1_, all stimuli (bradykinin, methacholine and exercise), baseline symptom, inhaled corticosteroid dose, tests sequence and intercept were considered as covariables. All the models are fitted by the method of maximum likelihood using the SAS software 9^th ^version [14].

## Results

We studied 20 asthmatic outpatients (10 women; age range: 21 – 46 years). All subjects were on inhaled short-acting β_2_-agonists as rescue medication and 14 subjects on inhaled corticosteroid therapy. Characteristics of studied subjects can be seen in Table 1. The geometric mean PC_20 _MCh was 0.36 (range 0.08 to 2.35 mg/ml), and the geometric mean PC_20 _BK was 0.68 (range 0.05 to 3.87 mg/ml). The mean (± SD) FEV_1 _fall after exercise was 20.45% ± 3.43%.

**Table 1:** Characteristics of the subjects studied

Subject	Gender	Age (years)	BMI	FEV_1_ (L)	FEV_1_ (%)	Tests sequence	PC_20 _MCh (mg/ml)	PC_20 _BK (mg/ml)
1	M	23	23.8	3.24	76	BK, EIB, MCh	0.83	2.30
2	M	23	24.2	3.56	83	EIB, BK, MCh	0.21	0.15
3	M	21	24.0	2.98	69	BK, EIB, MCh	0.19	1.16
4	F	30	19.1	3.44	110	MCh, EIB, BK	1.13	3.87
5	F	43	24.8	2.55	83	EIB, MCh, BK	0.56	0.42
6	F	23	25.8	2.26	68	MCh, BK, EIB	0.13	0.58
7	F	45	25.7	2.13	80	EIB, BK, MCh	0.47	1.62
8	F	39	27.9	1.66	54	MCh, BK, EIB	0.14	0.12
9	F	24	33.6	2.81	87	BK, MCh, EIB	0.16	1.17
10	F	38	31.2	2.56	88	BK, MCh, EIB	1.00	0.22
11	M	39	22.1	2.90	74	EIB, MCh, BK	0.62	0.06
12	F	24	32.7	3.16	91	MCh, EIB, BK	0.17	1.16
13	M	24	19.2	3.73	90	EIB, MCh, BK	0.42	1.48
14	M	27	31.8	3.26	77	MCh, BK, EIB	0.28	2.37
15	M	46	26.8	3.29	83	EIB, BK, MCh	0.38	0.71
16	M	33	23.1	3.21	73	BK, MCh, EIB	2.35	2.32
17	F	24	19.6	2.57	80	MCh, EIB, BK	0.95	3.56
18	F	26	21.8	2.30	80	MCh, EIB, BK	0.15	0.20
19	M	38	24.6	2.25	64	BK, MCh, EIB	0.08	0.05
20	M	32	28.7	3.00	80	MCh, BK, EIB	0.58	1.73

Mean		31	25.5	2.84	79.5		0.36^a^	0.68^a^

SD		8	4.4	0.54	11.6		2.45^b^	3.84^b^

M: male; F: female; BMI: body mass index; FEV_1_: Forced expiratory volume in one second; MCh: methacoline; BK: bradykinin; EIB: exercise-induced bronchospasm; PC_20_: provocative concentration that results in a 20% fall in FEV_1_.

^a ^geometric mean. ^b ^geometric standard deviation.

Table 2 shows intensity of symptoms among stimuli. Cough induced by bradykinin was more intense. Bradykinin also led to more intense breathlessness detected by VAS scale, but not by Borg scale. The pairwise comparisons of challenge tests are shown in Table 3. For the cough evaluation, bradykinin caused more cough in comparison with methacholine and exercise (p < 0.01). There were no differences between exercise and methacholine (p = 0.31). In terms of dyspnea measured by Borg scale, there were no differences among stimuli (p ≥ 0.15). When dyspnea was measured by VAS, bradykinin induced more dyspnea than exercise (p = 0.04) and than methacholine (p = 0.02).

**Table 2:** Symptoms intensity during bronchoconstriction

Stimuli	Cough (total of episodes)	Dyspnea – Borg	Dyspnea – VAS
Bradykinin	72.40 ± 69.26*	3.60 ± 2.30	3.26 ± 2.47#
Methacholine	18.15 ± 20.58	3.40 ± 2.40	2.94 ± 2.32
Exercise	5.95 ± 8.22	2.10 ± 2.00	2.12 ± 2.05

Mean ± Standard Deviation.

* # Significant differences (p < 0.05) in comparison with other stimuli according to linear mixed-effects model.

**Table 3:** Pairwise comparisons according to linear mixed-effects model of modified Borg scale, visual analogue scale (VAS) and cough (logarithmic scale).

Cough			
Comparisons	Mean Difference*	95% CI	p-value

Bradykinin × Exercise	2.79	(1.83; 3.76)	< 0.01
Bradykinin × Methacholine	2.22	(1.12; 3.32)	< 0.01
Exercise × Methacholine	-0.58	(-1.69; 0.53)	0.31

Dyspnea – Borg			

Comparisons	Mean Difference*	95% CI	p-value

Bradykinin × Exercise	1.20	(-0.41; 2.81)	0.15
Bradykinin × Methacholine	0.59	(-1.23; 2.43)	0.52
Exercise × Methacholine	-0.60	(-2.46; 1.25)	0.52

Dyspnea – VAS			

Comparisons	Mean Difference*	95% CI	p-value

Bradykinin × Exercise	1.76	(0.09; 3.42)	0.04
Bradykinin × Methacholine	2.28	(0.39; 4.18)	0.02
Exercise × Methacholine	0.53	(-1.39; 2.44)	0.59

CI: confidence interval. *The model was adjusted by intercept, age, gender, atopy status, body mass index, FEV_1_, baseline FEV_1_, bradykinin, methacholine and exercise, baseline symptom, inhaled corticosteroid dose and tests sequence.

The baseline FEV_1 _had effect on cough (estimate 3.10; 95%CI 0.77–5.43; p < 0.01) and on dyspnea evaluated by Borg scale (estimate 6.96; 95%CI 2.95–10.96; p < 0.01) or by VAS (estimate 5.08; 95%CI 0.89–9.26; p = 0.02). These positive-estimate values indicate that higher baseline FEV_1 _leads to more symptoms during FEV_1 _fall. For the following covariables, no effect has been detected on cough or dyspnea: body mass index, atopy status, age, gender, tests sequence and inhaled corticosteroid dose.

## Discussion

In this cross over study, 20 asthmatic subjects were evaluated. Self-reported ratings of the intensity of dyspnea (assessed by Borg and VAS) and coughing counts were made during bradykinin, methacholine and exercise induced bronchoconstriction. The study showed that bradykinin induced more cough and dyspnea than methacholine and exercise. As expected, dyspnea increased during challenge tests, so did cough. Some studies failed to show this dose-related increase in cough counts during bronchoconstriction [15,16].

Our data support the proposal of using cough to evaluate bronchial responsiveness in special groups of patients. Technical difficulties in performing spirometry are common: one out of five elderly subjects cannot perform spirometry according to the international guidelines [17]. Similarly, approximately 30% of pre-school children are unable to perform acceptable efforts [18]. In the evaluation of challenge induced cough, bradykinin would probably be the best stimulus because it could increase the sensitivity of the test by increasing cough intensity.

In addition, during challenge tests, cough and dyspnea were significantly more intense in subjects with higher baseline pulmonary function (baseline FEV_1_). Probably, patients with prolonged airflow obstruction would be less breathless for any given reduction in FEV_1 _than those with higher baseline FEV_1_, a process known as temporal adaptation [19]. This is in favor of the use of cough to define positive bronchial challenge test, given that most patients who need challenge tests are not severe enough to have very low FEV_1_.

Some theories are able to explain why, in patients with asthma, the severity of breathlessness is greater during bradykinin than methacholine or exercise challenge at given levels of airway obstruction: a) bradykinin challenge test causes more cough and this could interfere in dyspnea perception, increasing it; b) methacholine has PC_20 _lower than bradykinin and this could be associated with faster bronchospasm, shorter sensory duration of the experience; c) intensity of asthma symptoms depends on the mechanisms that are involved in the induction of airway obstruction [10]. The difference in mode of action among three stimuli should be considered potential determinant of breathlessness severity. For instance, after exercise, various sensations from physical discomfort could have influenced scoring of perceived breathlessness and it is not known what the effect of exercise itself is on cough or sensation of dyspnea. One solution to these potential problems could be a bronchial provocation challenge that would reproduce the hyperventilation of exercise, e.g. eucapnic voluntary hyperventilation of cold and dry air [20].

Another study has showed that bradykinin inhalation caused cough and retroesternal discomfort, but authors did not evaluate cough quantitatively [16]. In a more recent study with 12 subjects with mild asthma, authors recorded and counted coughs episodes. They showed that, in general, bradykinin induced more coughing than did methacholine, however, there were some subjects who rarely coughed to either stimuli, whereas others had a marked cough response regardless of the stimuli [15].

Despite cough is a very common symptom and the mechanisms contributing to it are widely studied, there has been much debate, for instance, surrounding the identity of the airway afferent nerve subtype that precipitates reflex coughing. Studies in experimental animals and in humans show clearly that multiple afferent nerve subtypes (mechanosensors and chemosensors) might be involved in the production of reflex coughing. However, not all stimuli evoke cough under all conditions. This might suggest divergence between multiple reflex pathways or the existence of primary and secondary cough afferent pathways [21]. Also, there is a suspicious that a complex allergic reaction in the airway may be involved in the development of antigen-induced increase in cough reflex sensitivity [22]. There is evidence of the involvement of airway vagal afferents, such as sensory C-fibers, and rapidly adapting receptors in the cough reflex, as well as in other symptoms of respiratory disease, such as bronchospasm [23,24]. Bradykinin, capsaicin and citric acid, stimuli that are known to active airway chemosensors, are amongst the most potent tussigenic agent in conscious animals and humans[21].

The mechanisms of tussive and bronchoconstrictor responses to bradykinin may be the same, via C-fibers [25]. The non-myelinated C-fibers contain the tachykinins substance P, neurokinin A and neurokinin B which, upon release, act on NK1, NK2, NK3 receptors respectively to mediate several functions [26]. Whilst inhalation of citric acid stimulates both C-fibers and rapidly adapting receptors, capsaicin appears to stimulate only C-fibers and both these agents have been shown to induce cough, in several species including man, and also bronchoconstriction [21,26-30].

In several studies, dyspnea score are usually plotted against percentage of fall in FEV_1 _and individual symptoms/FEV_1 _ratios are used to represent an index of dyspnea, and their corresponding intercepts, representing baseline symptoms. These variables are calculated by linear regression analysis [19,31]. The mixed procedure fits a variety of mixed linear models to data and enables the use of these fitted models to make statistical inferences about the data. The linear mixed-effects models, therefore, provides flexibility of modeling not only the means of data (as in the standard linear model) but their variances and covariances as well [14].

Some studies make video recordings or employ simultaneous recordings of flow rate, air volume, subglottic pressure and acoustic signal to evaluate cough. However, the use of different devices could interfere with dyspnea sensation, plus, there is a recent study (comparing video recordings and audio recordings) showing that trained observers are able to achieve good agreement counting cough manually from audio recordings [32]. Another study showed that the agreement between simultaneous (at the same time when the test is being conducted) and video counting of coughs is generally good. To ensure reliable simultaneous cough counting, challenge tests should be performed in a quiet environment, applying as little unnecessary equipment and measurements as possible [33].

## Conclusion

Bradykinin challenge provides the strongest coughing intensity and breathlessness for a given fall in FEV_1_, and is thereby recommended for protocols planned to evaluate cough. Bradykinin may act on neural mechanisms that modulate symptoms, increasing cough and dyspnea in asthmatic patients. These data corroborate previous studies that showed, in experimental models, a role for bradykinin in the mechanism of cough.

## List of abbreviations

FEV_1_: forced expiratory volume in one second; VAS: Visual Analogue Scale; PC_20_: provocative concentration of any stimulus resulting in a 20% fall in FEV_1_; BK: bradykinin; MCh: methacholine; EIB: exercise-induced bronchospasm; NK: neurokinin.

## Competing interests

The authors declare that they have no competing interests.

## Authors' contributions

TRS recruited the subjects, performed the data collecting and draft the manuscript. EZM and CAG performed the statistical analysis and interpretation of data. EOV participated in conception, design of the study, coordination, helped to draft the manuscript and critical revision. All authors have given final approval of the version to be published.
